# Identification of putative domain linkers by a neural network – application to a large sequence database

**DOI:** 10.1186/1471-2105-7-323

**Published:** 2006-06-27

**Authors:** Satoshi Miyazaki, Yutaka Kuroda, Shigeyuki Yokoyama

**Affiliations:** 1 Department of Biophysics and Biochemistry, Graduate School of Science, University of Tokyo, 7-3-1 Hongo, Bunkyo-ku, Tokyo, 113-0033, Japan; 2 RIKEN Genomic Sciences Center, 1-7-22, Suehiro-cho, Tsurumi, Yokohama 230-0045, Japan; 3 Department of Biotechnology and Life Science, Graduate School of Technology, Tokyo University of Agriculture and Technology, 2-24-16, Nakamachi, Koganei, 184-8588, Tokyo, Japan

## Abstract

**Background:**

The reliable dissection of large proteins into structural domains represents an important issue for structural genomics/proteomics projects. To provide a practical approach to this issue, we tested the ability of neural network to identify domain linkers from the SWISSPROT database (101602 sequences).

**Results:**

Our search detected 3009 putative domain linkers adjacent to or overlapping with domains, as defined by sequence similarity to either Protein Data Bank (PDB) or Conserved Domain Database (CDD) sequences. Among these putative linkers, 75% were "correctly" located within 20 residues of a domain terminus, and the remaining 25% were found in the middle of a domain, and probably represented failed predictions. Moreover, our neural network predicted 5124 putative domain linkers in structurally un-annotated regions without sequence similarity to PDB or CDD sequences, which suggest to the possible existence of novel structural domains. As a comparison, we performed the same analysis by identifying low-complexity regions (LCR), which are known to encode unstructured polypeptide segments, and observed that the fraction of LCRs that correlate with domain termini is similar to that of domain linkers. However, domain linkers and LCRs appeared to identify different types of domain boundary regions, as only 32% of the putative domain linkers overlapped with LCRs.

**Conclusion:**

Overall, our study indicates that the two methods detect independent and complementary regions, and that the combination of these methods can substantially improve the sensitivity of the domain boundary prediction. This finding should enable the identification of novel structural domains, yielding new targets for large scale protein analyses.

## Background

Structural genomics/proteomics projects seek to establish high-throughput techniques by promoting routine protein structure determination either by X-ray crystallography or NMR spectroscopy [1-7]. However, the determination of large protein structures remains as a major hurdle, especially for NMR, which requires elaborate techniques and time consuming analyses [8]. Even when X-ray crystallography is employed, the average size of proteins determined by this method and listed in the PDB (Protein Data Bank) is about 230 residues. This situation not only reflects the difficulty of determining large protein structures, but also that of expressing and purifying them. Meanwhile, most large proteins are assembled from structural domains, which are structurally independent units that are able to fold into a native structure even when isolated from the rest of the protein. Thus, dissecting large proteins into their structural domains can provide several candidates for swift structural analysis by either X-ray crystallography or NMR spectroscopy.

Protein dissection is often a long and tedious process. Limited proteolysis is the prevalent experimental method for determining structural domain boundaries [9-12], but it does not alleviate the problems related to the expression and purification of large proteins. Screening methods for detecting natively folded proteins without relying on a specific functional activity have recently been developed [13,14], and they may serve as tools to isolate natively folded domains from a library of randomly generated protein fragments, thus alleviating the need to first purify the full length protein. However, experimental methods are usually time-consuming, and less expensive computer-aided methods for detecting putative domains in protein sequences have practical values for all types of high-throughput proteomics projects [15].

Various theoretical methods for identifying domains in protein sequences have recently been reported. These include well-established sequence similarity searches against existing domain databases, such as Pfam or SMART [16-19]. A major limitation of these methods is their inherent inability to identify completely novel domains. On the other hand, methods that do not rely on a pre-existing domain database can be valuable tools in high-throughput structural genomics projects as they can identify novel, natively folded domains suitable for structural analysis[20,21]. Thus, the prediction of domain organization based on sequence information alone is presently an actively investigated topic [22].

Recently, domain prediction methods based on sequence information alone, such as the statistics of residue contact in domains [23], the statistics of domain size distribution [24], the sequence characteristics of domain linkers [25-27], the amino acid composition of domain linkers [28-30], covariance analysis [31]and the conservation of hydrophobic clusters [32] have been developed. Some of the aforementioned methods to detect domain boundary sequence characteristics use neural networks [25-27]. Neural networks [33] have been successfully applied to the prediction of several aspects of protein structure, such as secondary structures [34,35], β turns[36], structural classes[37], and stabilization centers[38], but its use in domain boundary recognition is relatively new [25].

In this paper, we used our neural network [25] to search for putative domain linker regions in the SWISSPROT database [39]. The aim of the present study was threefold. First, we asked if our neural network – which was trained with a small data set of 74 multi-domain proteins derived from SCOP [40] – could be applied to a practical problem, specifically, that of detecting protein domains for structural genomics/proteomics projects from a large sequence dataset. Second, we were interested in comparing our predictions, which rely only on sequence characteristics, with traditional methods that detect domains by sequence similarity to domain databases; here, we used the Protein Data Bank (PDB) [41] and the Conserved Domain Database (CDD) [19]. Last, we examined the possibility of improving the detection of domain boundaries by combining the detection of the putative domain linkers with that of the low-complexity regions, which encode unstructured protein sequence segments. Overall, the present analysis confirmed our previous study, and indicated that our neural network can efficiently detect domain boundaries even when applied to a large and "real" sequence database.

## Results and discussion

### Detection of putative domain linkers by the neural network

In many applications, including ours, it is critical to reduce the number of false positives because of their experimental costs, while false negatives are not as detrimental. In our neural network, a 'cutoff' parameter determines the balance between specificity and sensitivity (i.e., the balance of false positives and false negatives) [25]. Thus, we searched for putative domain linkers in 101602 SWISSPROT sequences using high cutoff values, ranging from 0.90 to 0.98, to minimize false predictions even at the cost of missing existing linkers. The number of putative domain linkers identified by our neural network ranged from 1469 to 20876 for cutoffs of 0.98 and 0.90, respectively. As expected, the use of a higher cutoff parameter increased the number of correct predictions, but decreased the total number of predicted domain linkers (Table 1). Overall, the same conclusions are reached independently from the cutoff value, when it is between 0.90 and 0.98. The following discussion is based on a search with a cutoff value of 0.95, which yielded 8133 putative domain linkers, representing 1.4% of the data set on a residue number basis (Table 1). These figures correspond to approximately one putative linker predicted for every 12 sequences, which is a tractable number for a high-throughput experiment.

### Assignment of 'putative structural domains'

For the purposes of this discussion, we define 'putative structural domains' as sequence segments with high similarity to PDB or CDD sequences (sequence identity >30% and sequence overlap > 85%; See details in the Material and methods section). Putative structural domains are thus able to fold into a native structure or at least to form a domain, and we used them to assess the correctness of the predicted domain boundaries. As anticipated, a substantial fraction of the SWISSPROT sequences is covered by known putative structural domains. Specifically, from a total of 101602 SWISSPROT sequences, 38470 sequences (corresponding to, respectively, 38% and 27% on a sequence and residue basis) had similarity to a PDB sequence, and 64349 sequences (43% on a residue basis) had similarity to a CDD sequence (Table 1).

### Correlation between predicted linkers and putative structural domain termini

**Figure 1:**
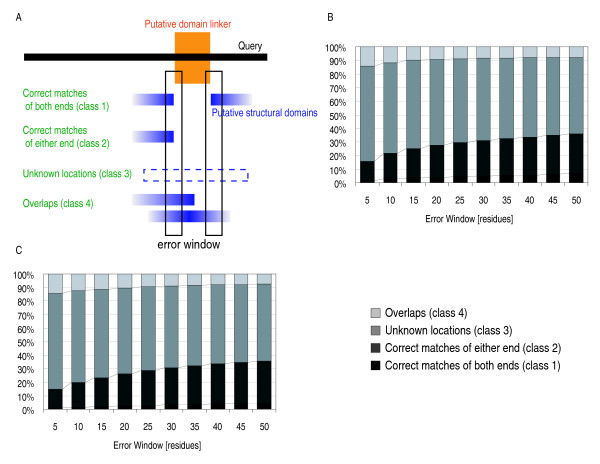
Classification of the predicted linkers and the low complexity regions. (A) Schematic representation of the positions of the predicted domain boundaries relative to the putative structural domains. The our classes are: correct matches at both ends (class 1), correct matches at either end (class 2), overlaps (class 4), and unmatched locations(class 3). Percentages of putative domain linkers (B) and low-complexity regions (C) in the four classes. An error window parameter, on the horizontal axis, is used to accommodate the terminal ambiguity of the assigned sequence regions. When the distance between the ends of a putative domain linker (B) or a low-complexity region (C), and the end of a putative structural domain was smaller than the error window, we considered the position of the predicted domain boundary to be correct. The error window parameter was varied from 5 to 50 residues.

Our method for evaluating the correctness of the predicted domain linkers was to assess their positions relative to those of putative structural domains. To this end, we classified the putative domain linkers into four classes (Figure 1A; see Materials and methods). Linkers that matched either one or both ends of a putative structural domain were classified into classes 1 and 2, respectively, and were considered as correctly predicted. Putative domain linkers overlapping with putative structural domains are likely to break them in two non-foldable sequences. They were thus counted as incorrect predictions, and classified in class 4. Finally, putative linkers that were located far away from any putative structural domains (farther than the error window discussed below) were categorized in class 3. These linkers could not be evaluated as either correct or incorrect.

The putative structural domains as defined above may contain multiple structural domains, and, hence, some linkers in class 4 may be correctly located. Our calculations thus slightly underestimate the actual performances of both the neural network and the LCRs predictions (see also next section). However, the underestimations are likely to be very small, and concern only a few percents of the putative linkers, as most proteins in the PDB (and many in the CDD) are single structural domain proteins [28,29].

**Figure 2:**
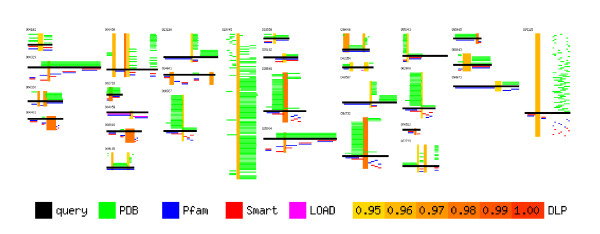
Putative domain linkers and low-complexity regions assigned in SWISSPROT sequences. Each thick black horizontal bar represents a SWISSPROT sequence used as a test sequence. The SWISSPROT ID number is indicated on the top left of the corresponding sequence. In each SWISSPROT sequence, sequence regions similar to PDB and CDD sequences were assigned as putative structural domains. A green horizontal bar represents a sequence region similar to a PDB sequence. Similarly, the horizontal bars colored in blue, red and magenta represent sequence regions similar to CDD sequences, corresponding to the Pfam, SMART and LOAD (Library Of Ancient Domains) libraries, respectively. Sequence regions predicted to be putative domain linkers are designated by vertical bars in colors ranging from yellow to brown, according to the neural network output values. Low-complexityregions are designated by cyan rectangles overlaid on black bars.

The above classification was performed by allowing an error window between the position of the predicted linker and the termini of the putative structural domain. As expected, when the error window was increased, the occurrence of correct matches increased while that of the overlaps decreased. With an error window of 20 residues, the percentages of correct matches (classes 1 and 2), overlaps (class 4) and unknown locations (class 3) were 27.5%, 9.2% and 63.4%, respectively (Figure 1B). Thus, 75% of the putative domain linkers with predictions that could be evaluated (classes 1, 2 and 3) were correctly located, suggesting that the boundaries of the putative structural domains can be predicted with reasonable confidence. On the other hand, almost two-thirds of the putative domain linkers were predicted in regions without a corresponding putative structural domain nearby, possibly delimiting novel structural domains not yet classified in the PDB or CDD (Figure 2).

### Detection of low-complexity regions

Most large-scale sequence databases contain a substantial number of long, unstructured, disordered regions that may interfere with systematic searches for structural domains. Thus, the detection of unstructured portions of proteins as defined by low complexity regions (LCRs), which are unlikely to fold into a globular structure [42], or structurally disordered regions [43] may help predict domain boundaries, although this was not the original intent. Here, we examined whether LCRs as detected by SEG [42], overlapped with domain boundaries. Two parameters in the SEG program, called trigger and extension complexity, control the balance between the detection number (Table 1) and the ratio of correct matches relative to incorrect ones (data not shown). In order to analyze approximately the same number of sequences as that of the putative linkers detected with the cutoff of 0.95, we set the trigger complexity to 2.9 and the extension complexity to 3.2, which yielded 8539 low-complexity regions (Table 1). Using an error window of 20 residues, the percentages of correct matches (classes 1 and 2), overlaps (class 4) and unknown locations (class 3) were 26.3%, 10.3% and 63.4%, respectively (Figure 1C). Thus, the position of the LCRs correlate with the temini of the putative structural domains at a level similar to that observed for the domain linkers (Figure 1B).

### Comparison of domain boundaries detected by domain linkers and LCRs

**Figure 3:**
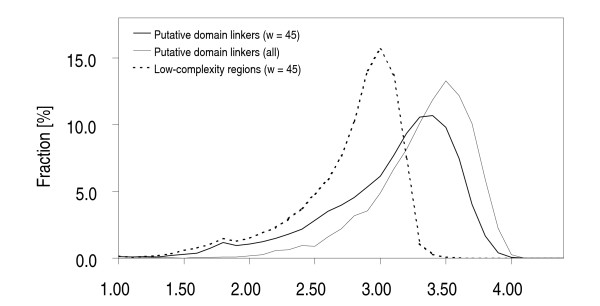
Complexity distribution. The sequence entropy distributions are shown for the putative domain linkers (thick solid line) and the low-complexity regions (thick broken line) longer than 45 residues. The sequence entropy was calculated by a sliding window of 45 residues over the putative domain linkers [43, 51]. The thin solid line represents the sequence entropy of all of the putative domain linkers (including those shorter than 45 residues) calculated with a window equal to the length of the linker.

Although both the domain linker and LCR predictions correlate well with the putative structural domain termini, it is important to note that the LCRs and linkers are located in different sequence regions. Indeed, only 2561 out of 8539 LCRs overlapped with the putative domain linkers predicted by our neural network, and, in turn, 2643 out of 8133 putative linkers were detected by the SEG program (Table 2). Furthermore, the sequence entropy of the putative linkers was higher than that of the LCRs, with the maximum of the sequence entropy distribution at around 3.5 for the linkers, while it was only 3.0 for the LCRs (sequence complexity values lower than 2.9 are unlikely to fold into a globular structure). Thus, our neural network appears to detect preferentially non-globular regions with higher sequence complexity than those detected by SEG. These results indicate that LCRs and linker sequences have different characteristics, and that the two methods are complementary for identifying domain boundaries (Figure 3).

As a result of their complementarity, the sensitivity of the domain detection was clearly improved by combining the LCR and linker predictions (Table 1; Figure 3). A combined search yielded 13946 domain boundaries, i.e., only 2726 sequences less than the total of the LCR and linker sequences. Furthermore, the domain boundary sequences identified by a combined LCR-linker search were categorized into the 4 classes in percentages similar to those identified by the separate LCR and linker searches. Thus, the total number of correctly predicted domain termini increased 1.6 fold, while the fraction of incorrect predictions (false positives) remained unchanged.

### Comparison with random guesses

**Figure 4:**
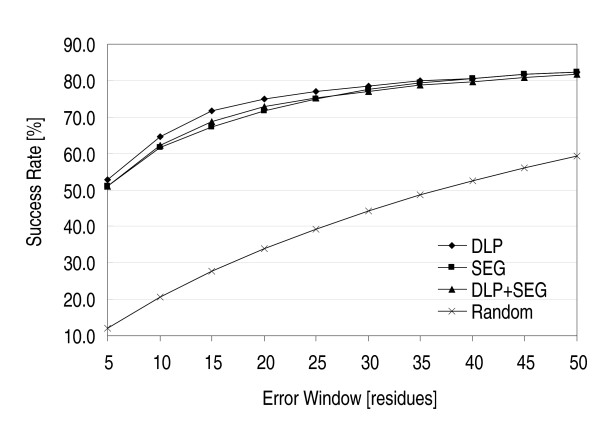
Comparison with blind prediction. The success rate (prediction quality index) of blind prediction is plotted as a function of the error window parameter (cross marks). The prediction quality factors for domain linkers (diamonds), low-complexity regions (squares), and a combined prediction (triangles) are also shown.

As a further assessment of both our neural network and the SEG program to detect putative structural domain termini, we estimated the success rate of a blind prediction. The blind prediction was defined as the probability that a randomly assigned residue in the query sequence matches with a putative structural domain terminal residue within the allowed error (Materials and methods). We compared the random guesses with our neural network and SEG prediction using a quality index calculated as the ratio of correct predictions relative to the sum of correct and incorrect predictions [44-46], which is computed as the number of sequences in classes 1 and 2 divided by those in classes 1, 2 and 4. Figure 4 clearly shows that the quality index of the blind prediction is far below those of the two other methods. This result strongly supports our initial assumption that the occurrences of both the putative domain linkers and the low-complexity regions near the putative structural domain terminal regions are not fortuitous.

### Domain termini and error windows

**Figure 5:**
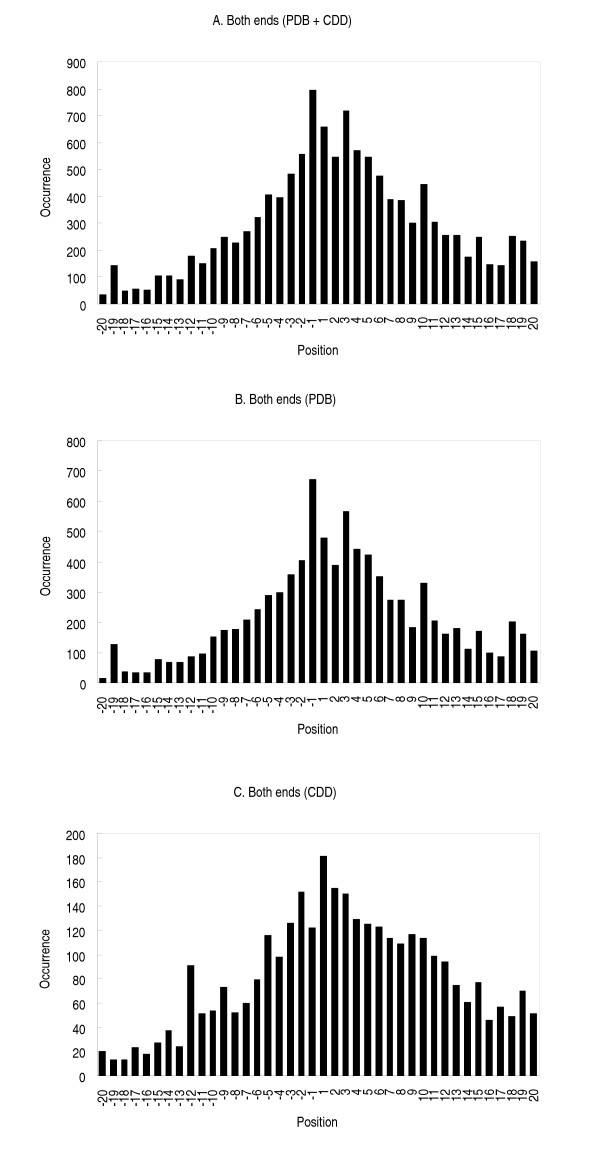
Correlation between the positions of domain linkers and putative structural domains. The horizontal scale represents the number of residues in the error window between the linker termini and the corresponding putative structural domain termini. This is calculated as the number of residues separating the last residue (or the first residue) of a domain linker in Classes 1 and 2 from the first residue (or respectively the last residue) of the corresponding putative structural domain. (A) Distribution calculated for putative structural domains detected by similarity to PDB and CDD, (B) to PDB, and (C) to CDD.

From a practical viewpoint, it is important to evaluate the error window within which the boundaries are predicted. The exact position of a domain boundary is obviously ambiguous. The first reason is that PDB sequences may include several unstructured terminal residues (without coordinates), causing some uncertainties about the exact positions of the putative structural domain termini. The uncertainty arising from the CDD sequence is even larger. Second, the smoothing windows used to reduce the spurious predictions introduce ambiguity in the positions of the predicted domain linkers, as they smear their C and N termini. These issues can be examined using an error window parameter that accommodates the positional ambiguity generated by both the putative structural domain termini and the predicted domain linkers (or LRCs). As shown in Figure 5, the positions of the first and last residues of the predicted domain linker are distributed randomly around the positions of the last and respectively first residue of the structural termini. This shows that the error distribution is random with a maximum at 0 residue, confirming that the linker positions are accurately assigned. The error is clearly limited to about 20 residues, and to 10 residues in most cases. Furthermore, the prediction quality index dependence on the error window also indicates that the ambiguity is limited to about 20 residues, as it reaches 70% for a 15 residue error window and then rapidly levels off for larger windows (Figure 4).

## Conclusion

Our study strongly suggests that sequence characteristics alone, as detected by either our neural network or SEG, can identify domain boundaries in protein sequences even without sequence similarity to existing domain databases. There is a clear correlation between the termini of putative structural domains and the positions of both the domain linkers and the LCRs. Furthermore, our neural network and SEG are complementary for detecting domain boundaries, and when combined, the sensitivity of the domain boundary prediction is increased without decreasing its specificity. Overall, our study shows that domain identification protocol based on domain boundary prediction can be applied to practical problems, such as the identification of novel structural domains, and thus will yield new targets for large scale protein analyses.

## Methods

### Sequence databases and estimation of the putative structural domains

A total of 101602 SWISSPROT protein sequences [39] were used in the present investigation. Since the putative structural domains needed to be structurally independent units, we located all of the sequences with high similarity to PDB [47] and CDD [19] sequences, using the BLAST and RPS-BLAST programs[48,49]. To ensure the structural identity, as much as possible, we required a sequence identity greater than 30% and a sequential overlap greater than 85% over the entire length of the corresponding PDB or CDD sequence. Thus, putative structural domains detected by similarity to a PDB sequence are likely to fold into a structure similar to the corresponding PDB structure. Analogously, putative structural domains detected by similarity to CDD sequences, which is a compilation of conserved protein domain sequences imported from Pfam [18] and SMART [16], are likely correspond to a natively folded domain, although their structures have not necessarily been determined.

### Putative domain linkers predicted by the neural network

We used a two hidden units neural network [50] trained to distinguish between domain linker and non-linker regions. The prediction procedure was identical to that reported in our previous paper [25], except for the following two points. (1) The prediction was carried out over the entire protein sequence, namely from the start to the end of each target sequence, because the SWISSPROT sequences may contain unstructured termini. Indeed, in our previous study, we assumed that a 60 residue length is the minimum for a polypeptide to fold independently, and we omitted the 60 terminal residues of the multi-domain protein sequences from the prediction, because the protein structures were known, and we knew that no unstructured termini were present. (2) Predicted domain linkers were not ranked, because under the stringent conditions (cutoff 0.90–0.98; see below) examined here, the prediction success rate was sufficiently high without such a procedure.

The smoothing window size and the threshold parameters were fixed to 19 and 0.5, respectively, as in our previous study. However, we set the cutoff parameter to values ranging from 0.90 to 0.98, because a high cutoff yields a better prediction specificity at the cost of the prediction sensitivity. The specificity and sensitivity for the first ranked domain linkers predicted with a cutoff of 0.90 are 81.8% and 10.3%, respectively, as calculated with a ten-fold jack-knife [25].

### Low-complexity regions

Sequence entropy (also called Shannon's entropy) has been used to quantify the complexity of amino acid sequences, and several studies have examined the relationship between the sequence entropy and the globularity of proteins [42,43]. According to these studies, the sequence entropy of globular proteins is generally high, with a lower limit of around 2.9.

SEG is a program that identifies low-complexity regions in protein sequences [51]. This program was originally intended to distinguish between globular and non-globular regions. In this study, we used SEG to check whether a correlation between the low-complexity regions and the putative structural domain termini existed. Three parameters in SEG, the trigger window length, the trigger complexity and the extension complexity, are used to assign low complexity regions. We set the trigger window length to 45 residues, in line with previous studies [43,51] To obtain a number of LCRs similar to that of the linkers predicted with a cutoff of 0.95, the trigger and extension complexities were set to 2.9 and 3.2, respectively (Table 1 and Figures 1 and 3).

### Evaluation of putative domain linkers and low-complexity region

We evaluated the validity of the prediction of the domain boundaries from their positions relative to the putative structural domains as defined above. The predicted domain boundaries were divided into four classes (Figure 1A), using an error window to accommodate the ambiguity in the termini position of both the predicted domain boundaries and the putative structural domains. A predicted domain boundary was considered to be correctly located when its end was separated from a putative structural domain by fewer residues than specified by the error window (Figure 1A). Class 1 includes predicted domain boundaries in which the closest ends are located within the error window of a putative structural domain. Predicted domain boundaries with both ends located within the error window of the N and C terminal ends of two putative structural domains are categorized in class 2. Class 3 consists of predicted domain boundaries that are separated from any putative structural domain by a number of residues larger than the error window.

### Random guess

We assumed the success rate of a blind prediction, *i.e*. a prediction without any *a priori *information, to be the probability that a randomly assigned position matches a terminal residue of a putative structural domain. Four classes were defined similarly to those used to evaluate the putative domain linkers and the low-complexity regions. For example, a randomly picked residue was considered to be correctly located and was classified in class 1, when the end of a putative structural domain was found within the error window. The success rates (quality index) for the blind prediction, the putative domain linkers and the low-complexity regions were calculated as the rate of correct matches (classes 1 and 2) relative to both the correct and incorrect matches (classes 1, 2 and 4).

## Authors' contributions

S.M. designed the study, wrote the programs, analyzed the data, and wrote the paper under the supervision of Y.K. Y.K. conceived the study, analyzed the data and wrote the paper with S.M. S.Y. supervised S.M. and the study.

## Figures and Tables

**Table 1:** 

Sequence regions detected	No. of sequences^a^	No. of sequence regions^b^	No. of residues^c^	% residues^d^
All	101602		37315215	100.00
PDB	38470	410090	10210325	27.36
CDD	64349	124888	16207467	43.43
Low-complexity regions (45, 3.4, 3.75)^e^	48641	70373	8474412	22.71
Low-complexity regions (45, 2.9, 3.2)	6735	8539	803001	2.15
Low-complexity regions (45, 2.6, 2.9)	3208	3970	359227	0.96
Low-complexity regions (45, 2.45, 2.75)	2340	2786	250796	0.67
Putative domain linkers (0.90)^f^	14239	20876	1051607	2.82
Putative domain linkers (0.91)	12670	18193	953097	2.55
Putative domain linkers (0.92)	11160	15620	856149	2.29
Putative domain linkers (0.93)	9554	13053	752119	2.02
Putative domain linkers (0.94)	7977	10591	644472	1.73
Putative domain linkers (0.95)	6387	8133	529884	1.42
Putative domain linkers (0.96)	4819	5892	415150	1.11
Putative domain linkers (0.97)	3099	3592	281009	0.75
Putative domain linkers (0.98)	1326	1469	128455	0.34
Low-complexity regions (45, 2.9, 3.2) + Putative domain linkers (0.95)^g^	10364	13946	1139983	3.06

Statistics of SWISSPROT sequences. ^a ^Number of SWISSPROT sequences that contained the detected sequence regions. ^b ^Number of sequence regions detected in the SWISSPROT sequences. ^c ^Total number of residues in the detected sequence regions. ^d ^Percentage of residues in the detected regions relative to all of the residues in the SWISSPROT sequences. ^e ^The values of the three parameters used for the SEG program, namely, the trigger window, the trigger and extension complexities are listed in the parentheses. ^f ^The cutoff parameter used for our neural network is indicated in the parentheses. ^g ^Predictions obtained by merging putative domain linkers and the low-complexity regions.

**Table 2:** 

	Putative domain linkers^a^		Low-complexity regions^b^	
	Uniquely linker regions	Overlapped with low-complexity regions	Overlapped with putative domain linkers	Uniquely Low complexity
Correct matches of both ends (class 1)	236 (4.3%)	94 (3.6%)	97 (3.8%)	101 (1.7%)
Correct matches of either end (class 2)	1241 (22.6%)	665 (25.2%)	706 (27.6%)	1358 (22.7%)
Unknown locations (class 3)	3469 (63.2%)	1684 (63.7%)	1544 (60.3%)	3851 (64.4%)
Overlaps (class 4)	544 (9.9%)	200 (7.6%)	214 (8.4%)	668 (11.2%)
Total	5490	2643	2561	5978

Overlaps between putative domain linkers and low-complexity regions. ^a ^The putative domain linkers were assigned by the neural network with a cutoff of 0.95. ^b ^The low-complexity regions were assigned by the SEG program with a trigger window of 45 residues, a trigger complexity of 2.9, and an extension complexity of 3.2.
